# Transdermal Delivery of Succinate Accelerates Energy Dissipation of Brown Adipocytes to Reduce Remote Fat Accumulation

**DOI:** 10.1021/acs.molpharmaceut.2c00628

**Published:** 2022-10-25

**Authors:** Fang-Hsuean Liao, Chun-Nien Yao, Shu-Ping Chen, Te-Haw Wu, Shu-Yi Lin

**Affiliations:** Institute of Biomedical Engineering and Nanomedicine, National Health Research Institutes, 35 Keyan Road, Zhunan Town, Miaoli County 35053, Taiwan

**Keywords:** succinate, microneedle delivery, brown adipose tissue, energy dissipation, obesity, proton leak

## Abstract

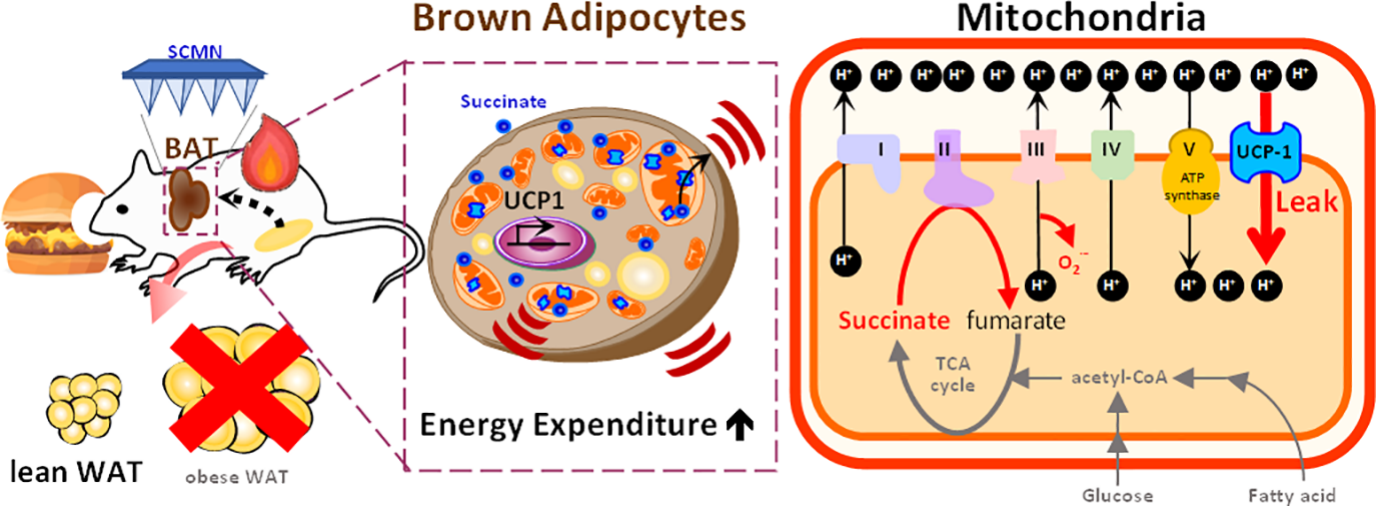

Weight loss by increasing energy consumption of thermogenic adipocytes to overcome obesity remains a challenge. Herein, we established a transdermal device that was based on the local and temporarily controlled delivery of succinate (SC), a tricarboxylic acid cycle metabolic intermediate to stimulate the thermogenesis pathway of uncoupling protein 1 (UCP1) and accelerate energy dissipation of brown adipose tissue (BAT) under the dorsal interscapular skin, further initiating the consumption of fatty acids by systemic metabolism. SC microneedle patches significantly suppressed weight gain and fat accumulation of remote organs, including liver and peripheral white adipose tissue (WAT) in high-fat diet-induced obese mice. mRNA expression levels of *Ucp1* in BAT and other browning markers in WAT were significantly elevated in the mice that were treated with SC microneedle. Thus, the energy dissipation of BAT using UCP1-mediated thermogenesis accelerated by the transdermal delivery of SC may become a potential and effective strategy for preventing obesity.

## Introduction

Brown adipose tissue (BAT) is considered thermogenic adipocytes that burn calories to perform energy consumption and dissipation, whereas white adipose tissue (WAT) is regarded as fat depots to store the excess energy.^1,2^ Even though the biological strategy has been found to transform the subcutaneous WAT to inducible thermogenic adipocytes (also called brown-like-in-white or beige adipocytes),^3,4^ the energy expenditure of beige adipocytes is dominated by cold-inducible thermogenesis that is not commonly used to fight obesity.^5,6^ Increasing thermogenesis of brown adipocytes through the energy dissipation process of calorie expenditure might be an efficient approach to fighting obesity. Still, it is unclear whether the fat storage of WAT can be decreased by BAT thermogenesis. It is generally known that the thermogenesis of brown adipocytes requires activation of cyclic AMP-protein kinase A (cAMP) by external stimuli, such as β3-adrenergic stimulation through the sympathetic nervous system^7^ or cold exposure.^8^ However, oral β3-adrenoceptor agonists place a heavy burden on the cardiovascular system.^9,10^ Currently, other therapeutic strategies, including the implantable wireless device and local delivery of chemical compounds as external stimuli, have been reported to induce beige adipocytes by targeting the subcutaneous white fat of mice,^11−13^ but these methods have not been used to augment the BAT thermogenesis.

Succinate (SC), the mitochondrial tricarboxylic acid (TCA) cycle intermediate, is required for the energy conversion of mitochondrial electron transport chain (ETC) through participating hydrogen ions (protons) across the inner mitochondrial membrane (from matrix to intermembrane space), leading to a gradient potential of mitochondrial protons. Finally, the high electrochemical proton gradient is used by the adenosine triphosphate (ATP) synthase (namely, complex V) to generate ATP through oxidative phosphorylation in most cells or by uncoupling proteins to leak protons back to the mitochondria matrix that consequently cause energy dissipation as heat in brown adipocytes.^14,15^ The inner mitochondrial membrane of brown adipocytes is rich in uncoupling protein 1 (UCP1) that can make protons leak back to the matrix, leading to thermogenesis.^15^ Previous studies reported that, during blood circulation, orally ingested systemic SC was taken up by brown adipocytes,^16^ bypassed ETC complex I,^17^ fueled ETC complex II activity,^18^ and triggered the membrane potential leading to noncanonical fat thermogenesis for energy expenditure.^16^ However, systematic administration of SC has a high risk for intestinal inflammation.^19,20^ Therefore, an alternative method of local delivery of SC directly targeting or directly stimulating brown adipocytes would be a potential strategy against obesity development.

**Figure 1:**
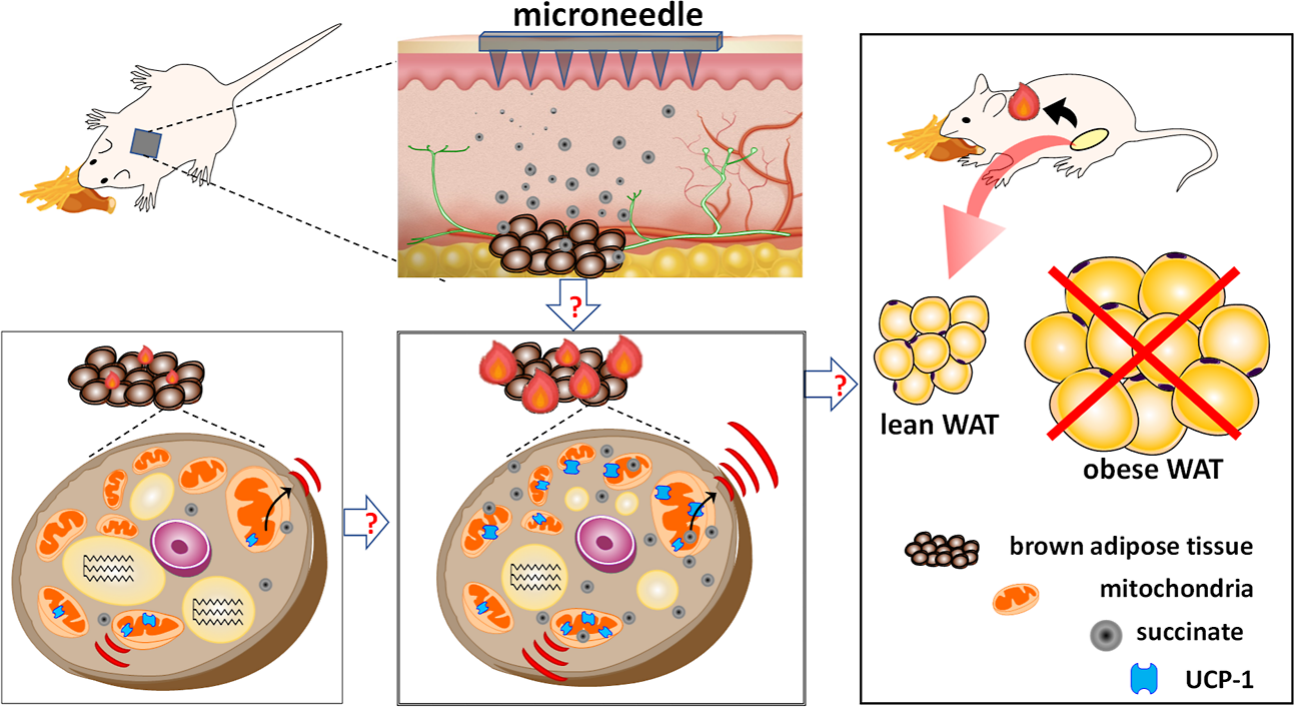
Transdermal device to deliver SC as a stimulator of thermogenic adipocytes was proposed. A putative illustration summarizes that SCMN may sufficiently target BAT to increase energy dissipation and reduce the fat accumulation of remoted organs.

In the study, we aimed to address the local and temporary delivery of SC to BAT that may be able to accelerate energy dissipation of brown adipocytes and therefore reduce remote fat accumulation, and the concept is shown in Figure 1. To do this, we used a dissolvable polymeric microneedle system that has been shown to increase skin permeability, which might be suitable for the encapsulation of biotherapeutic agents and small-molecular SC.^21−23^ It is known that hyaluronic acid (HA) is mainly present in the epithelial cells and the extracellular matrix of tissues and is regularly degraded by hyaluronidases.^24^ With its biocompatible and biodegradable properties, we use HA to fabricate a SC-encapsulated microneedle (SCMN) for local transdermal delivery as an alternative to exogenous SC uptake. Additionally, the application of HA microneedle (HAMN) arrays might also use a lower effective dose of SC that is rapidly dissolved (within a few minutes) when inserted into the skin.^22,25^ Another advantage is that a painless and bloodless microneedle could reduce infection during the delivery of SC through the skin depth.^26,27^

## Materials and Methods

### Chemicals and Reagents

All chemicals, including sodium SC (S9637), oligomycin, carbonyl cyanide p-(trifluoromethoxy) phenylhydrazone (FCCP), rotenone, antimycin-A, triiodo-l-thyronine (T3), indomethacin, 3-isobutyl-1-methylxanthine (IBMX), dexamethasone, and insulin, were obtained from Sigma-Aldrich (Saint Louis, MO, USA). HA (MW 8000–15000) was purchased from CARBOSYNTH (Berkshire, UK). Fluorescent dye Cy5.5-NHS was purchased from the Lumiprobe Corporation (Hunt Valley, Maryland, USA). Bone morphogenetic protein 7 (BMP7) was purchased from R&D Systems (Minneapolis, MN, USA). Primary antimouse UCP1 (#GTX112784) and F4/80 (#ab6640) antibodies were purchased from GeneTex (Irvine, CA, USA) and Abcam (Cambridge, UK), respectively.

### HAMN-Encapsulated SC

A part of the aqueous solution containing 30 wt % HA (MW 8000–15 000, CARBOSYNTH) and 3.3 wt % disodium SC (Sigma) (0.3 g ∼ 0.4 g) was taken to mix with SC. Mixed solution was put into a polydimethylsiloxane (PDMS) mold to centrifuge at 2000*g* rpm for 5 min at room temperature. After centrifuging, the PDMS mold was taken out and placed under normal pressure and room temperature overnight until the microneedle dried. The SCMN patch was taken out from the PDMS mold and stored in a vacuum box at room temperature prior to use. The SC weight percentages of SCMN patches were estimated to be 3.291% for each batch.

### Scanning Electron Microscope

The appearance of microneedle patches was observed using a Hitachi TM1000 Tabletop Scanning electron microscope (SEM, Hitachi, Europe GmbH, Krefeld, Germany).

### Animal Care and Experimental Procedures

Male 12-week-old C57BL/6JNarl mice were purchased from the National Laboratory Animal Center (Taipei, Taiwan) and were housed in a controlled environment with a 12:12 light–dark cycle, moderated humidity, and temperature under specific pathogen-free conditions at the Laboratory Animal Center of the National Health Research Institutes (NHRI). The animal center is accredited by the Association for Assessment and Accreditation of Laboratory Animal Care International (AAALAC International). All experimental animal procedures used followed published guidelines approved by the Institutional Animal Care and Use Committee of NHRI (the approval code: NHRI-IACUC-108135-A November 2019). Mice were fed a commercial HFD (60% energy from fat, D12492; Research Diets, Inc., New Brunswick, NJ, USA) for a week of acclimatization period and 7 weeks of the experimental period. During the HFD feeding acclimatization period, the hair on the dorsal skin of all mice was removed using depilatory cream (Nair, Church & Dwight Co, Princeton, NJ). Shaved mice were randomly assigned to the two groups with different delivery routes of SC.

SC water group (SCW) had free access to drinking water containing 2% sodium SC for oral delivery. SC was loaded in a degradable HAMN patch (SCMN) for transdermal delivery. Once under anesthesia, mice in the SCMN group received microneedle patches on the right dorsal skin above interscapular BAT depots for 5 min and were kept under a warm lamp to prevent hypothermia during anesthesia, twice a week for 7 weeks. Twice-weekly administration frequency was based on previous studies for applying drug-loaded microneedle patches to the subcutaneous inguinal WAT of mice.^12,13^ Body weight, drinking water, and food were regularly monitored. At the end of the experiment, all mice were euthanized by isoflurane overdose. Serum was collected, and organs were harvested, weighed, cut into several small pieces for histological staining or for RNA extraction, and quickly frozen in liquid nitrogen. All samples were stored at −80 °C until analysis.

### In Vivo Imaging System for Microneedle-Delivered Animal Imaging

Anesthetized mice received microneedle patches containing encapsulated Cy5.5 on the right upper site of dorsal skin for different amounts of time. The Cy5.5 fluorescence signal was monitored using the IVIS Imaging System 200 Series (PerkinElmer Inc.).

### Hematoxylin and Eosin Staining and Immunohistochemical Staining

Tissues were fixed by fresh 10% formaldehyde overnight and then underwent tissue processing for paraffin embedding, and 5 μm-thick sections were prepared by the Pathology Core Laboratory of NHRI. The tissue sections were stained with hematoxylin and eosin (H&E) to check cellular and tissue features. Hematoxylin precisely stains nuclear components a purplish blue, while eosin stains cytoplasmic components pink. The sections were also immunostained with primary antibodies specific to UCP1 and F4/80 following the standard protocol. Histological images were captured using an automatic digital slide scanner Pannoramic MIDI with a Plan-Apochromat 20×/0.8 objective (3D HISTECH Ltd., Budapest, Hungary) by the Pathology Core Laboratory of NHRI.

### Adipocyte Size

To quantify the adipocyte size, images of H&E-stained histological sections of inguinal and epididymal WATs from mice were captured by Pannoramic Viewer at 10× magnification. At least 500 adipocytes per mouse were measured by cellSens dimension desktop software (Olympus). These images were also measured, and the frequency distribution of the adipocyte sectional area was calculated.

### Blood Analysis

Serum SC levels were determined by EnzyChrom SC assay Kit (#ESNT-100, BioAssay Systems, Hayward, CA, USA). Serum cytokine IL-6 and TNF-a levels were measured by commercial mouse ELISA kits (BioLegend, Inc., San Diego, CA, USA). Serum free fatty acid (FFA) was measured using a FFA quantification colorimetric kit (Biovision, Milpitas, CA, USA).

### Quantification PCR

Adipocyte tissues were prehomogenized in TRIzol with Biomasher disposable homogenizers and centrifuged at 4 °C to remove particulate debris, and the supernatant was transferred into an RNase-free tube. Next, RNA isolation was performed using the Direct-zol RNA MiniPrep kit (Zymo Research, Irvine, CA, USA) according to the manufacturer’s recommendation and quantified by Nanodrop (Thermo Fisher Scientific Inc., Waltham, MA, USA). 1 μg of RNA was reversely transcribed into complementary DNA (cDNA) using the maxima first-strand cDNA synthesis kit (Thermo Fisher Scientific Inc., Waltham, MA, USA) by a PCR machine (Mastercycler X50s; Eppendorf, Hamburg, Germany). For gene transcription, cDNA templates were amplified for 40 cycles in the FastStart Universal SYBR Green Master (ROX) kit (Roche, Mannheim, Germany) with 300 nM primers (Table S1) specific to genes of the interest by a LightCycler 480 thermocycler (Roche, Mannheim, Germany). Relative expression was normalized to the housekeeping gene *36B4* (acidic ribosomal phosphoprotein P0) in adipocyte samples and calculated using 2^–ΔΔCt^ methods.^28^

### Cell Culture

Immortalized mouse brown preadipocyte WT-1 cell line was purchased from Millipore-Sigma (# SCC255, Temecula, CA, USA). WT-1 cells were maintained in high-glucose Dulbecco’s modified Eagle’s medium (DMEM) medium supplemented with 10% heat-inactivated FBS, 584 mg/L l-glutamine, 3.7 g/L sodium bicarbonate, 110 mg/L sodium pyruvate, 100 U/mL penicillin, and 100 μg/mL streptomycin. Cells were incubated at 37 °C, 5% CO_2_, and 20% O_2_ in a humidified incubator. For differentiation, WT-1 cells were differentiated into mature brown adipocytes as described previously with minor modifications.^29^ Briefly, WT-1 cells were seeded on 10 cm dishes at a density of 3 × 10^4^ cells/cm^2^ in maintenance medium and allowed to attach overnight. Then, cells were cultured in the presence of 3.3 nM BMP7 in an induction medium containing 20 nM insulin and 1 nM triiodo-l-thyronine (T3, T6397, Sigma) in high-glucose DMEM supplemented with GlutaMAX (Gibco, Life Technologies), 2% FBS, and penicillin/streptomycin for 3 days. Cells were then exposed to a differentiation cocktail that consisted of 0.5 mM IBMX, 0.125 mM indomethacin, 5 μM dexamethasone, 20 nM insulin, and 1 nM T3 in DMEM medium for 2 days. On day 5, mature cells were refed with the induction medium without BMP7 for up to 8 days, and the medium was refreshed every other day.

### Permeabilized Cells

At the indicated day, mature brown adipocytes were pretreated with 0.75 nM plasma membrane permeabilizer (PMP, #102504-100, Agilent Technologies, Inc., Cedar Creek, TX, USA) in mitochondrial assay solution (MAS) for 25 min in the 37 °C incubator following the Seahorse XF PMP quick start guideline. PMP is a cholesterol-dependent cytolysin secreted by *Clostridium perfringins*, and it forms pores in the plasma membrane that permit SC into mitochondria and allow monitoring of SC-driven mitochondrial superoxide production and respiratory activity.^30^ MAS buffer (pH 7.2) consisted of 70 mM sucrose, 220 mM mannitol, 10 mM KH_2_PO_4_, 5 mM MgCl_2_, 2 mM *N*-(2-hydroxyethyl)piperazine-*N*′-ethanesulfonic acid, 1 mM EGTA, and 0.2% (w/v) fatty acid-free bovine serum albumin.

### MitoSOX Red Mitochondrial Superoxide Staining

Mature brown adipocyte WT-1 cells on day 8 were analyzed for SC-driven mitochondrial superoxide production. Before studying mitochondrial superoxide production, the medium was changed to freshen the induction medium without BMP7 for 1 day. Cells were suspended using 0.25% trypsin/ethylenediaminetetraacetic acid (EDTA) solution until the cell layer was dispersed, medium containing FBS to neutralized trypsin/EDTA solution was added, and then the cells were centrifuged to remove the supernatant. Cells were pretreated with 0.75 nM PMP in MAS buffer for 25 min in the 37 °C incubator and washed to remove the reagent. Resuspended, permeabilized cells at a density of 1 × 10^5^ per 100 μL MAS buffer in 1.5 mL tubes were treated with 5 mM SC in the presence or absence of 1 μM oligomycin for 30 min in the 37 °C incubator, washed, and then stained 5 μM MitoSOX for 10 min at 37 °C while protected from light. MitoSOX red superoxide indicator has maximum excitation and emission at 510 and 580 nm, respectively. Stained cells were analyzed using an Attune NxT acoustic focusing flow cytometer (Thermo Fisher Scientific), and flow cytometry data were analyzed using FlowJo software (v10, Tree Star).

### Mitochondrial Respiration

The oxygen consumption rate (OCR) was measured by a Seahorse XFe24 Analyzer (Agilent Technologies, Inc). On day 5, mature WT-1 cells were seeded on Seahorse XF24 cell culture microplate at a density of 16 500 cells per well and allowed to attach. On day 7, mature brown adipocytes were pretreated with 0.75 nM PMP in MAS buffer for 25 min in the 37 °C incubator following the Seahorse XF PMP quickstart guideline and the permeabilization protocol for the Seahorse assay as described by Salabei et al. (2014).^30^ In an OCR assay, permeabilized mature brown adipocytes WT-1 cells and all chemicals for OCR assay were maintained and prepared in MAS buffer. The protocol for running OCR was measured under basal conditions with three measurement cycles followed by the sequential addition of SC (5 mM) with eight measurement cycles, oligomycin (1 μM) with eight measurement cycles, FCCP (4 μM) with three measurement cycles, and a mixture of 0.5 μM rotenone and 0.5 μM antimycin A (Rot/AA) with three measurement cycles. In a SC-induced concentration-dependent OCR assay, the protocol was set under basal conditions with five measurement cycles followed by the sequential addition of low dose SC (0.1 mM) with seven measurement cycles and then high-dose SC (5 mM) with 30 measurement cycles. Each cycle of all assays was set to mix 1 min, wait 1 min, and measure 2 min to evaluate OCR.

### Statistical Analyses

For biologic assays, we used GraphPad Prism (v7.02) to perform unpaired, two-tailed Student’s *t*-tests, and one-way ANOVAs with Tukey’s multiple comparisons test. Data were expressed as mean ± standard error (SEM), and *p*-values of less than 0.05 were considered statistically significant.

## Results

### Skin Penetration of the Microneedle

**Figure 2:**
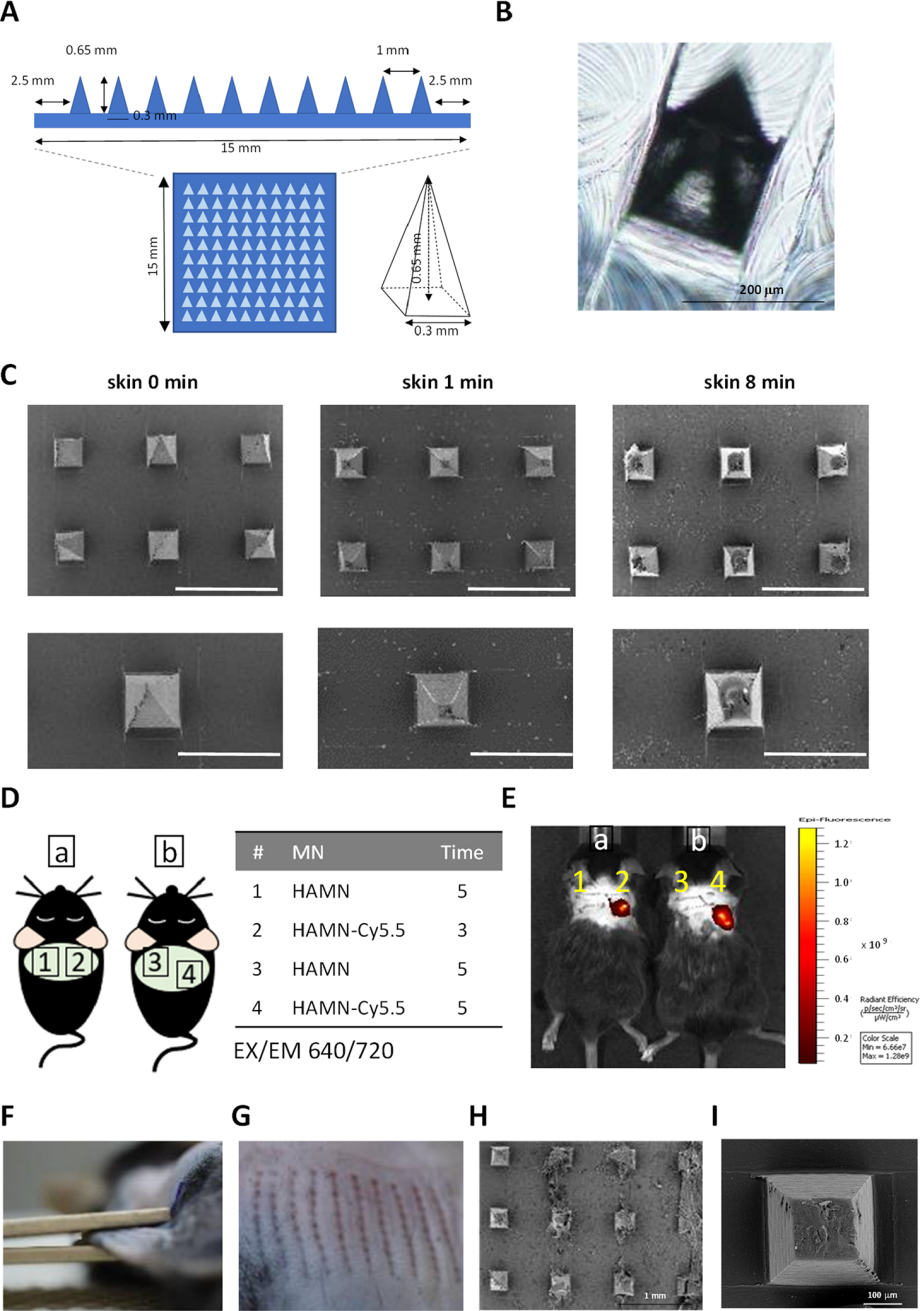
Characterization and skin penetration of microneedle was validated. (A) Detailed specifications of microneedle designs. (B) Optical microscope image showing a microneedle tip with a pyramid-shaped structure. (C) SEM photographs of a microneedle-loaded Cy5.5 applied on murine skin at the various indicated time points. (D) Illustration of microneedle application for examination in vivo for different times (sites 1 and 2) and with a warm lamp (sites 3 and 4). (E) IVIS results identifying the optimal microneedle application. (F) Illustration for application of microneedle treatment. (G) Representative murine dorsal skin after insertion. (H) SEM image of a microneedle patch after insertion into murine skin and (I) magnified SEM image of the single pillar.

The design feature of the SCMN has been reported in a previous literature with minor modifications (Figure 2A).^12^ Similarly, we requested a 15 × 15 mm microneedle patch consisting of a 10 × 10 array of microneedle tips with the base width of 300 μm and height of ≈650 μm. Each tip of the microarray was sculpted as a pointed and pyramidal shape with the aspect ratio of 2:1 from height to base diameter, which was indispensable for a drug injection microneedle proposed for skin. The tip-to-tip interspacing was 1 mm on the upper side of the microneedle designed for skin penetration.^27^ The optical miscopy (Figure 2B) showed that the microneedle tip had a sharp pyramidal point on the square basal structure, and the scanning electron microscope (SEM) images showed that each microneedle tip was very uniform (Figure 2C, the left column).

After successfully building the unique microneedle, we further examined the in vivo dissolution tests of microneedle-loaded Cy5.5 fluorescent dyes at the various indicated time points to monitor whether the structural integrity of the microneedle tips had been changed. As expected, the SEM images showed that more microneedle tips were dissolved in 8 min (Figure 2C, the right column) than in 1 min (Figure 2C, the middle column) after being inserted on murine skin. IVIS images showed that the microneedle encapsulated Cy5.5 fluorescent dyes were delivered into the murine skin of the interscapular region after 3 and 5 min (Figure 2D,E, site 2 and 4) compared with microneedle alone (Figure 2D,E, site 1 and 3). A stronger Cy5.5 fluorescent signal was observed when the skin was exposed to a mild heat lamp for 5 min (Figure 2D,E, site 4). After 5 min of microneedle application on skin (Figure 1F), the 10 × 10 array of microneedles had obviously penetrated the murine dorsal skin, and there was no evidence of damaged skin (Figure 2G). H&E staining formed a pit on the porcine skin (Figure S1). The SEM image of the microneedle patch used showed about 55% fracture (Figure 2H), and the single-pillar image confirmed a tip break (Figure 2I) after 5 min skin penetration. Accordingly, the attained insertion force was sufficient for skin penetration for drug delivery.

### Local SCMN Treatment Blunted Body Weight Gain and Fat Mass Accumulation of HFD-Fed Mice

HFD-fed mice were randomly assigned to two groups with different delivery routes of SC: the SCW and the SCMN treatment. The SCW group had free access to water containing 2% sodium SC, an effective dose reported to increase BAT thermogenesis.^16^ The SCMN group was received through transdermal delivery of SC twice a week. Here, we applied microneedle patches for local delivery of SC on the right upper site of dorsal skin because interscapular BAT depots can be found directly below this location.

**Figure 3:**
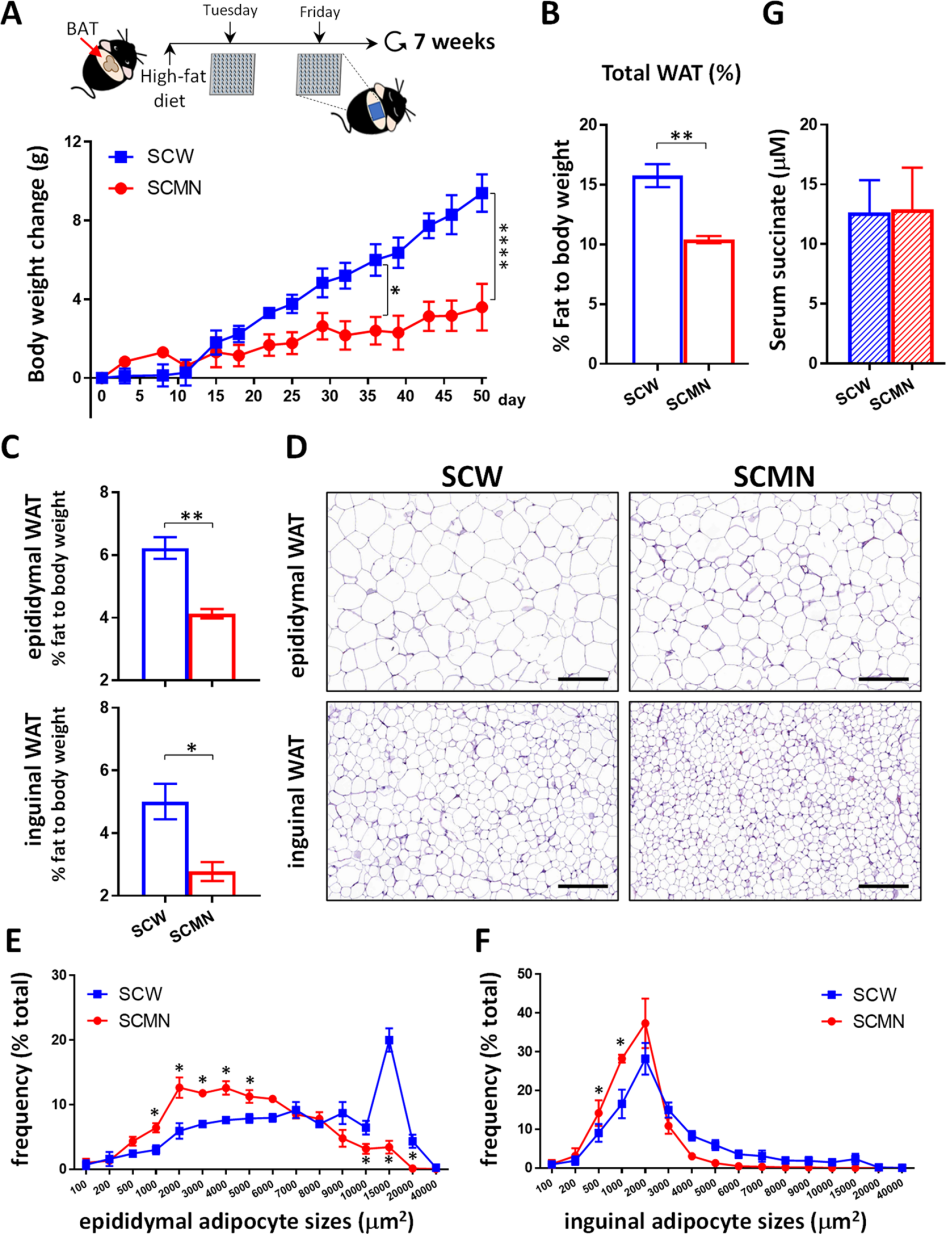
Local SCMN treatment suppressed body fat mass and shrunk the size of white adipocytes of HFD-fed mice. (A) Body weight changes of HFD-fed mice with different delivery routes of SC. (B) Percentage of total body fat against body weight. (C) Percentage of epididymal and inguinal WATs against the body weight of HFD-fed mice. (D) Representative images of H&E-stained sections of the epididymal WAT (upper panel) and inguinal WAT (lower panel) from mice. Scale bars, 200 μm. (E,F) Quantitative analysis of adipocyte size in the epididymal and inguinal WAT in (D), respectively. (G) Serum SC concentrations of mice with different treatments were measured. Data were expressed as mean ± SEM (*n* = 3–4). *p < 0.05, **p < 0.01 by unpaired, two-tailed Student’s *t*-tests.

Mice eating the HFD that received the SCMN treatment for 7 weeks (twice/week, 5 min/time) had significantly blunted body weight gain compared with the SCW treatment (Figure 3A). SCW and SCMN groups had a similar average daily food intake and water consumption (Figure S2). The result suggests that the total SC uptake of the SCMN group is lower than that of the SCW group because the microneedles were applied only twice per week. Analysis of body fat pads at necropsy (including epididymal, mesenteric, perirenal, and subcutaneous inguinal WAT) showed significantly less accumulation in mice with the SCMN treatment, leading to a ∼30% reduction of whole fat content compared to the SCW treatment (Figure 3B). This reduction was especially in epididymal and subcutaneous inguinal WAT mass with 30–40% reduction (Figure 3C) and in mesenteric WAT mass (Figure S3A), but not in perirenal WAT (Figure S3B).

We are also very interested in whether the microneedle treatment makes the mice feel stressed, which could change physiological demand. Thus, we prepared HAMN and plastic silicon microneedle (PSC) patches as another control group for HFD-fed mice to monitor body weight, food, and water intake. As expected, results showed that microneedle treatment did not cause reductions in food intake and body weight changes, while the PSC treatment caused the mice to consume less water than HFD-CTL (Figure S4A–D). Additionally, the HFD-fed mice treated with HAMN and PSC also exhibited no reduction in total body fat mass, visceral, or subcutaneous WAT (Figure S4E,F). Therefore, we can confirm the efficiency of the succinate microneedle (SCMN) patch and exclude any microneedle treatment stress that contributed to the body weight loss.

The local SCMN treatment impacted the size and morphology of white adipocytes of HFD-fed mice. Obesity is pathologically defined as being hypertrophic adipocytes, which means increasing cell size for storage of excessive fat from lipogenesis.^31^ Therefore, we sought to study whether SC administration by different delivery routes exhibits an impact on adipose morphology. H&E staining was used to confirm the adipose morphology of the visceral epididymal WAT and the subcutaneous inguinal WAT sections. Very interestingly, the cross-sectional area of histological images showed that epididymal and inguinal WAT of mice that received the SCMN treatment contained significantly smaller adipocytes than mice that received the SCW treatment (Figure 3D). These images were measured, and the frequency distribution of the adipocyte sectional area was calculated. Comparing the differences in the size distribution of the epididymal WAT of two groups, mice in the SCW group exhibited significantly greater numbers of large adipocytes (∼15 000 μm^2^) than mice in the SCMN treatment group, while mice with SCMN treatment had greater numbers of small fat cells (from 1000 to 6000 μm^2^, Figure 3E). Similar results were observed in subcutaneous inguinal WAT. The mice treated with SCMN had many more small adipocytes than mice treated with SCW (Figure 3F). Taken together, our results suggested that the local transdermal delivery of a small amount of SC through microneedles might target subcutaneous BAT and significantly magnify an antiobesity effect.

Given that elevating the systemic levels of SC has negative effects on host tissue,^19,20^ we further measured the blood SC to examine whether long-term local delivery of SC diffused to the whole body system of mice by the end of treatment. There was no difference in serum SC concentrations of mice between two groups with the oral administration or the local transdermal route (Figure 3G). The results implied that exogenous SC supplement did not affect homeostatic circulating SC, which was consistent with the previous reports.^16,32,33^

### Local SCMN Treatment Enhanced the UCP1-dependent Thermogenesis of BAT

**Figure 4:**
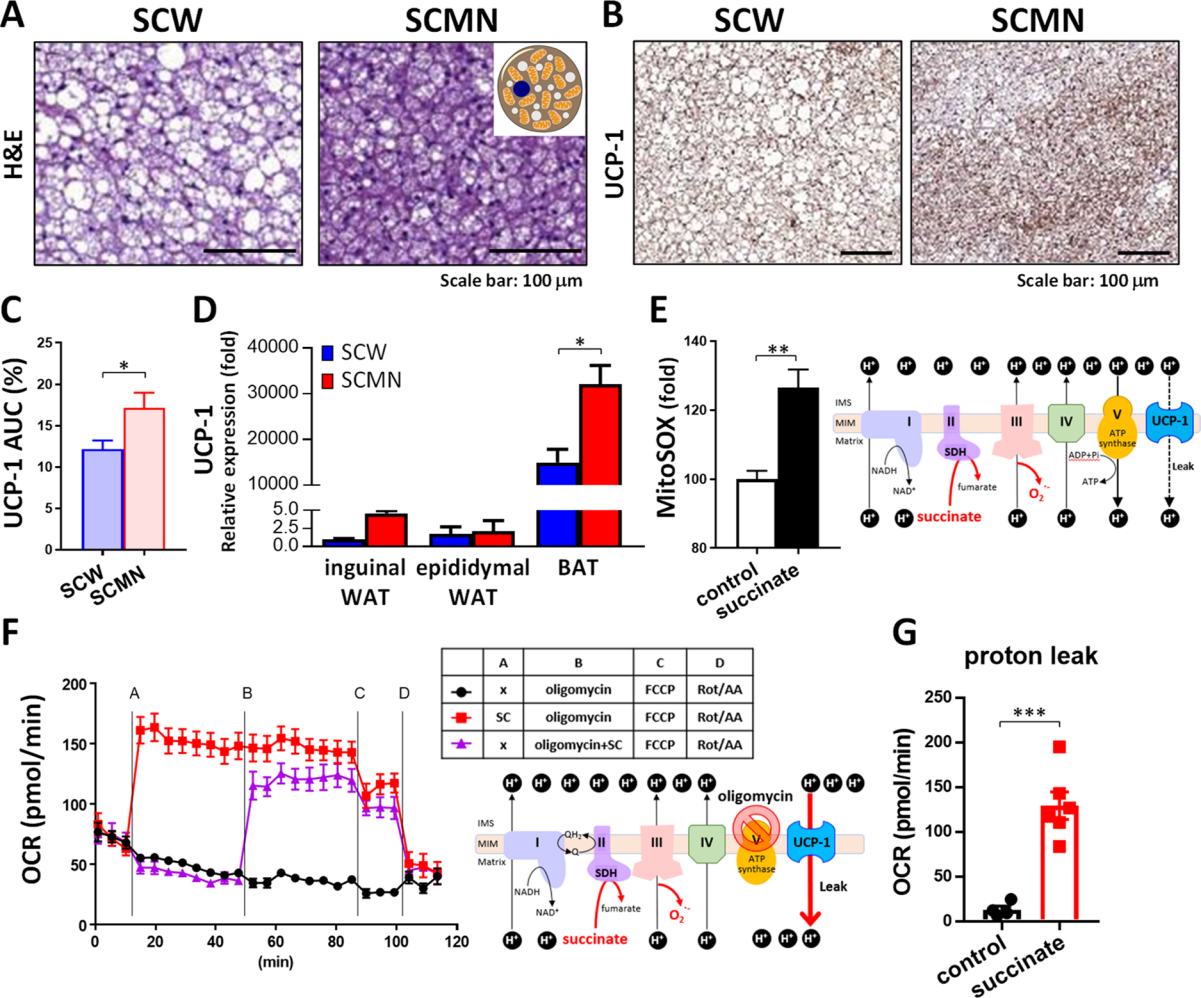
SC restored brown adipocyte features and accelerated mitochondrial proton leakage. (A) Representative images of the H&E-stained sections of the BAT from HFD-fed mice treated SCW or SCMN. Scale bars, 100 μm. (B) Immunohistochemical staining of UCP1 of BAT from mice with different treatments. Scale bars, 100 μm. (C) Quantification showing UCP1 expression of BAT from those mice. (D) RT-PCR analysis of UCP1 gene expression in BAT, epididymal WAT, and inguinal WAT of HFD-fed mice (*n* = 3–4). Relative mRNA expression was normalized to inguinal WAT of HFD-fed mice treated SCW. (E) Mitochondrial ROS levels in permeabilized mature brown adipocytes WT-1 cells treated with 5 mM SC for 30 min by mitoSOX staining with normalized control cells. The thin red line in the right cartoon panel indicates that SC induces ROS production. Abbreviations: IMS, intermembrane space; MIM, mitochondrial inner membrane. (F) OCR was determined using a Seahorse XF24 Analyzer under basal conditions and following the indicated addition of 5 mM SC, 1 μM oligomycin, 5 μM FCCP and a mixture of 0.5 μM rotenone and 0.5 μM antimycin A (Rot/AA) in permeabilized mature brown adipocytes WT-1 cells (*n* = 3–6 each experimental condition). The red line in the lower cartoon panel indicates that SC drives proton influx through UCP1. (G) Mitochondrial proton leakage was extensively increased in WT-1 cells in the presence of SC. Respiration of the proton leak was calculated as the difference between the addition of oligomycin and the addition of the Rot/AA mixture. Data were expressed as mean ± SEM. *p < 0.05, **p < 0.01, ***p < 0.001 by unpaired, two-tailed Student’s *t*-tests.

We further examined whether SC that entered brown adipocytes can show any difference in morphological alterations and UCP1 expression of BAT by different means of delivery. The H&E staining results showed that interscapular brown adipocytes of the SCMN group appeared to have more multilocular lipid droplets than those of the SCW group (Figure 4A). Magnification images of multilocular lipid droplets are shown in the top right corner of Figure 4A. Note, the expression of multilocular lipid droplets is emphasized as represented in a typical BAT feature.^2^ The finding illustrated that the microneedle delivery is very effective. Additionally, the IHC images revealed that SCMN produced high levels of UCP1 protein (Figure 4B,C). Consistently, higher UCP1 mRNA expression in BAT was found in SCMN-treated mice than SCW-treated mice (Figure 4D). Results also showed that increasing greater levels of UCP1 expression in BAT by microneedle delivery of SC was more effective than those in WAT.

Because SC also acts as a driver for reactive oxygen species (ROS) production,^16,34^ we next measured SC-driven mitochondrial ROS generation in differentiated WT-1 brown adipocytes with high UCP1 thermogenic gene expression.^29^ As expected, the SC treatment significantly increased the production of mitochondrial superoxide anions in WT-1 brown adipocytes (Figure 4E). However, WT-1 cells treated a combination with SC and oligomycin (ATP synthase blocker) and did not further boost mitochondrial ROS production compared with cells only treated with oligomycin (Figure S5). Therefore, we excluded that the reverse electron transfer of mitochondria to induce ROS was involved in the energy dissipation of WT-1 brown adipocytes. Our results instead suggest that there is an alternative pathway (such as UCP1) to reflux proton for oxygen respiration in mitochondria of brown adipocytes.

We further used the Seahorse XF analyzer to validate the important role of UCP1 in the mitochondria respiratory chain of brown adipocytes in the presence of SC and the potential effect of SC on mitochondrial UCP1-driven proton leak respiration. However, a diffusion-controlled intracellular influx of SC spent more than 1 h (Figure S6) could limit the OCR measurement window in intact (nonpermeabilized) WT-1 cells. Thus, we used perfringolysin O as a PMP that can increase the permeability of plasma membrane to permit SC influx into mitochondria to monitor mitochondrial respiratory activity.^30^ As measured as the OCR profile (Figure 4F), SC supplement in the mature brown adipocytes WT-1 cells with high UCP1 expression caused a dramatic increase in OCR values (Figure 4F red square line) compared to the control (Figure 4F black circle line). Also, simultaneous supplement of SC and oligomycin immediately increased mitochondrial OCR values (Figure 4F purple triangle line), indicating the continuous flow of proton leaks. To distinguish the contribution of two proton flux gates between mitochondrial oxidative phosphorylation (coupling respiration) by ATP synthase and UCP1-driven proton leak respiration (uncoupling respiration), oligomycin was used to block ATP synthase. Interestingly, we found that the OCR tracker of permeabilized WT-1 cells was insensitive to oligomycin treatment, which was similar to other permeabilized cells with a high degree of uncoupled respiration.^35^ After excluding the gate of ATP production by oligomycin, continuous SC supplement significantly augmented the mitochondrial proton leak 10-fold compared with the control in WT-1 cells without SC treatment (Figure 4G). Additionally, we also found that SC could induce a concentration-dependent increase of mitochondrial respiration, as shown in Figure S7. Taken together, the possible mechanism of transdermally delivered SC targeting BAT against obesity development was contributed to the acceleration of UCP1-dependent thermogenesis.

### Transdermal Delivery of SC Targeting BAT Increased Browning and Decreases Adipogenesis of WAT in HFD-fed Mice

The question of how the peripheral WAT of mice was used to augment the energy demand of thermogenic adipocytes due to local SCMN treatment was raised. We further examined whether the browning marker and lipid metabolism marker gene expressions have distinguishable differences in response to SC administration by different delivery routes. Analysis of browning marker gene expressions showed that the SCMN treatment significantly elevated expression of Cidea in inguinal WAT (Figure S8A), and the SCW treatment significantly increased PRDM16 in epididymal WAT (Figure S8B). Cidea contributes to WAT browning during cold exposure and controls the phenotype of lipid droplets from a unilocular to a multilocular form in subcutaneous WAT.^36^ Additionally, HFD-fed mice that received the SCMN treatment had significantly increased expression of adipose triglyceride lipase (a rate-limiting enzyme of lipolysis) in inguinal WAT (Figure S8C). Local SCMN treatment significantly blunted mRNA expressions of fatty acid synthase (FAS, the lipogenic marker) and fatty acid-binding protein 4 (FABP4, a mature adipocyte marker) and slightly increased mRNA expression of hormone-sensitive lipase (HSL, a lipolysis marker) in epididymal WAT of mice (Figure S8D). These results indicated that reduction in the size of WAT was primarily through decreased lipogenesis and increased lipolysis in response to transdermal delivery of SC. Therefore, decreased adipogenesis in WAT resulting from transdermally delivered SC targeting BAT could prevent the development of obesity.

### Local SCMN Treatment Decreased Systemic Inflammation in HFD-fed Mice

**Figure 5:**
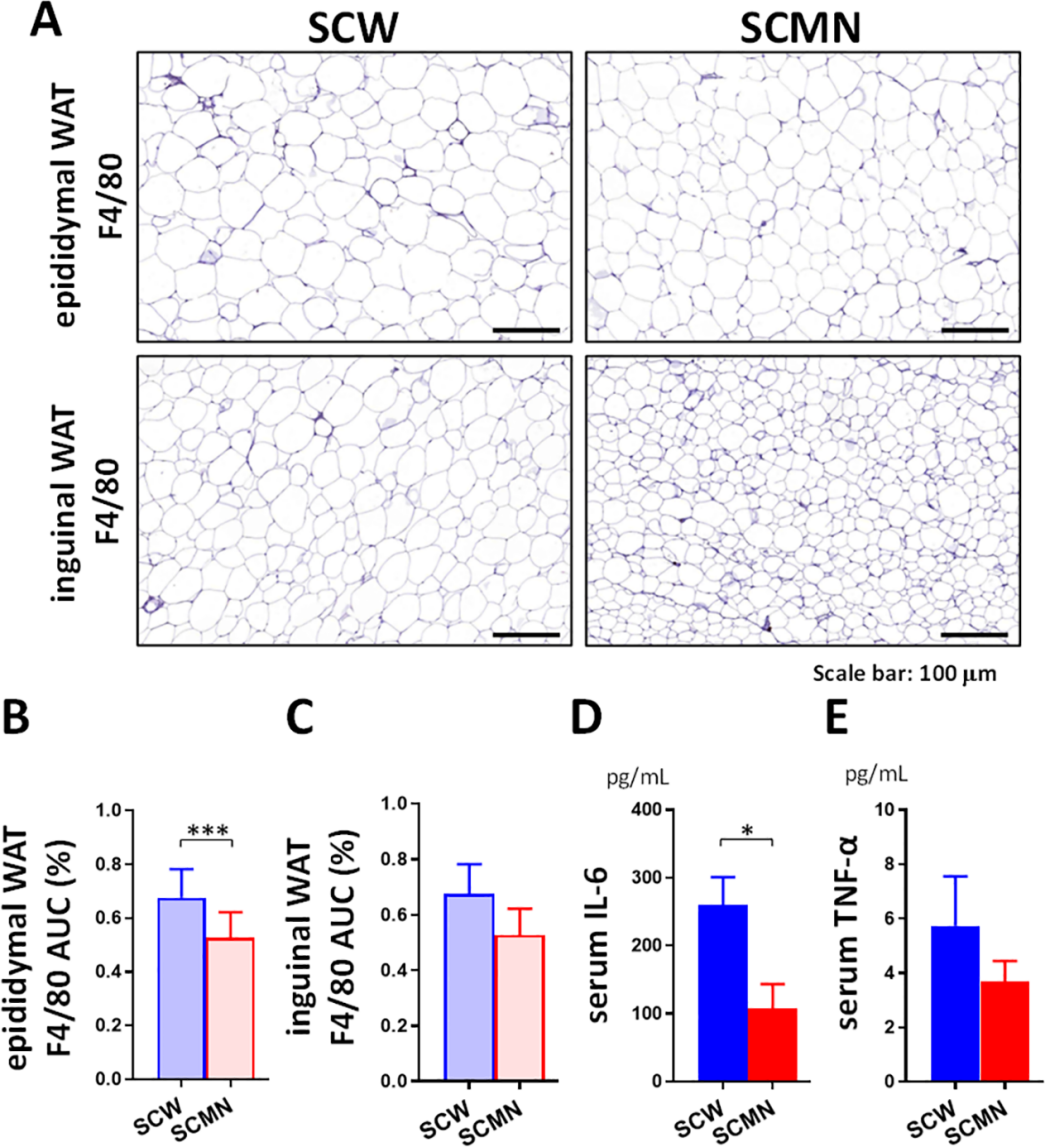
Local SCMN treatment reduced systemic inflammation in HFD-fed mice. (A) Representative images of H&E-stained sections of the macrophage marker F4/80 in inguinal and epididymal WAT from HFD-fed mice treated SCW or SCMN. Scale bars, 100 μm. (B,C) Quantification of F4/80 expression in inguinal and epididymal WAT from those mice. (D) Serum IL-6 and (E) serum TNF-α levels of mice were measured at the end of the experiment. Data were expressed as mean ± SEM (*n* = 3–4). *p < 0.05, ***p < 0.001 by unpaired, two-tailed Student’s *t*-tests.

Given that obesity is related to chronic low-grade inflammation due to increasing adipose macrophage infiltration and shifting macrophage polarization,^37^ we performed a histochemical analysis in which F4/80 was used as a pan marker to immunostain macrophages. Histological analyses revealed that HFD-fed mice that received the SCMN treatment had reduced macrophage infiltration in WAT (Figure 5A–C), consistent with a qPCR analysis of the marker F4/80 macrophage genes in WAT (Figure S9A,B). Moreover, the qPCR analysis revealed significantly higher mRNA expression of immunomodulatory M2-type macrophage marker genes *Arg1* and *Il-10* in the inguinal WAT of HFD-fed mice that received the SCMN treatment than in those mice that received the SCW treatment (Figure S9A). At the same time, expression of proinflammatory M1-type macrophage marker genes *Ccl2*, *Il-6*, and *Nos2* was significantly upregulated in epididymal WAT of obese mice treated with SCW (Figure S9B). These results suggest that the local SCMN treatment prevented HFD-induced adipose macrophage infiltration and modulated adipose macrophage polarization.

Next, the proinflammatory cytokines, including IL-6 and TNF-α, in serum from mice were examined after 7 weeks of treatments, even though the mRNA expression of TNF-*α* was significantly increased in inguinal WAT of HFD-fed mice that received the SCMN treatment (Figure S9A). Serum IL-6 concentrations were significantly increased by the SCW treatment in HFD-fed mice compared with the local SCMN treatment (Figure 5D), whereas changes in serum TNF-α concentrations failed to reach statistical significance between two groups (Figure 5E).

### Local SCMN Treatment Reduced the fat Accumulation of Remote Organs

**Figure 6:**
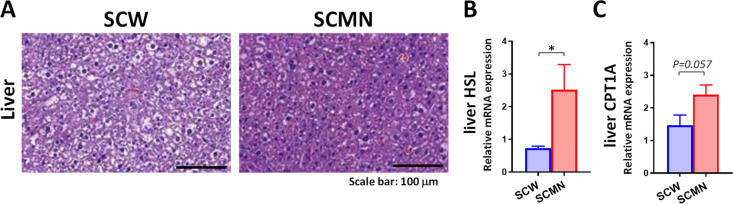
Local SCMN treatment alleviated HFD-induced fatty liver of mice. (A) Representative images of H&E staining showed the cytoplasmic changes in the hepatocytes from HFD-fed mice with different treatments. Scale bars, 100 μm. (B–C) Relative mRNA expressions of HSL and carnitine palmitoyltransferase 1A (CPT1A) in livers of obese mice with different treatments were measured. Data were expressed as mean ± SEM (*n* = 3–4). *p < 0.05 by unpaired, two-tailed Student’s *t*-tests.

Furthermore, the SCMN treatment reversed the cytoplasmic morphology of the vacuolated hepatocytes due to the HFD (Figure 6A), suggesting that SCMN treatment with BAT targeting is sufficient to reduce susceptibility to the development of HFD-induced fatty liver. We wonder if fatty acid accumulates in the blood circulation. Blood lipid profiles of obese mice were monitored, and no differences in serum TG (Figure S10A) or serum FFA (Figure S10B) concentrations were found between two groups. mRNA expression of hepatic HSL and carnitine palmitoyltransferase 1A (CPT1A, a key enzyme for transportation of fatty acids into the mitochondrion)^38^ was significantly increased by the local SCMN treatment (Figure 6B,C). The abovementioned results suggest that promoting brown adipocyte thermogenesis by SCMN treatment contributed to abolishing remote fat accumulation.

## Discussion

Currently, convincing evidence shows that the ablation of *UCP1* genes could obliterate BAT thermogenesis.^39^ Even though the UCP1 action mechanism still lacks direct evidence, UCP1 has been identified as acting as a proton-demand switcher in the mitochondrial membrane to release the energy of BAT.^40^ In particular, the level and activity of UCP1 are highly sensitive to the mitochondrial redox substances and carbon sources of TCA cycle, respectively.^41,42^ It is known that the ETC and the TCA cycles are closely coordinated metabolic processes.^14^ For supporting SC-driven ETC, the TCA cycle must use acetyl-CoA, derived from long-chain fatty acids and glucose breakdown, to require a steady supply of NADH and FADH_2_ (as electron carriers), which are essential to transfer electrons to the ETC. A pioneering study by Mills et al. demonstrated that long-chain fatty acids, as well as SC, were also taken up by brown adipocytes for increasing the UCP1-regulated energy expenditure.^16^ Long-chain FFAs, however, often stored in WAT and organs as lipid droplets, are required as an essential effector for the stimulation of UCP1 activity.^42−44^ We also observed the increased lipolysis in remote organs, such as WAT (Figure S8C,D) and liver (Figure 6B), of obese mice treated with SCMN. Thus, we speculated that our SCMN-delivery strategy might directly target BAT to stimulate the UCP1-regulated pathway, leading to an increase in the influx of FFAs into the mitochondrial matrix as a conformation ligand for increasing UCP1 activity, as a carbon source involved in the consumption of TCA cycle, and eventually forcing the energy dissipation of BAT. Therefore, the potential mechanism of a SCMN patch targeting BAT against obesity development contributed to the acceleration of UCP1-dependent energy dissipation, leading to the subsequent suppression of remote fat accumulation (Figure 6A).

Although antiobesity treatments, including diet control, aerobic exercise, bariatric surgery, and oral pharmacotherapies, have so far struggled to reverse obesity incidence or prevent recurrent obesity, increasing BAT thermogenic activity by cold stimulation and pharmacological-based treatments including β3-adrenergic receptor (β3-AR) agonists and mitochondrial uncoupling agents have been reported in human subjects and showed beneficial metabolic effects on insulin sensitivity.^45−48^ In addition, chemical lipophilic mitochondrial uncoupling agents, such as 2,4-dinitrophenol and BAM15, were used as antiobesity drugs to dissipate energy, but it has a narrow therapeutic window between effective and toxic doses and seems to be only for short-term use.^49,50^ Dietary SC itself is well tolerated; reports indicate that neither toxicity nor carcinogenic activity in F344 rats have been found after 2 years of continuous administration of 2% SC in drinking water.^51^

Human BAT is abundant in infants but was initially thought to atrophy and disappear during adulthood. However, a large human cross-sectional study has shown that plasma SC levels were negatively associated with total and visceral adiposity, indicating that SC links energy expenditure and brown adipocytes activation in humans.^52^ In most adults, a reservoir of brown preadipocytes is still present in the neck and upper chest that has been demonstrated by imaging tools and could be stimulated to recover its activation.^53^ A computational prediction tool found with almost 100% probability that both human and mouse classical BAT samples exhibited higher substantial thermogenic potential and displayed a 100-fold greater expression levels of UCP1 than beige adipocytes of subcutaneous WAT,^54^ which is consistent with the results of the RT-PCR analyses shown in Figure 4D. Our strategy based on SCMN treatment can efficiently blunt the state of HFD-feeding obesity via increasing the energy expenditure of BAT rather than direct browning of WAT. Therefore, we suggest that using transdermal delivery of SC to directly target BAT would be a feasible and effective strategy for obesity prevention in the future.

## Conclusions

In the study, we established that the local and temporary transdermal delivery of SC can efficiently blunt the state of HFD-feeding obesity via increased energy expenditure of brown adipose tissue, not only via direct browning of WAT. The simple strategy uses a transdermal device to locally deliver a TCA metabolic intermediate (SC) as a chemical stimulator that is able to accelerate energy dissipation of BAT under the dorsal interscapular skin, which leads to reducing the formation of distal WAT and eventually limits the fat accumulation of remote organs. Delivery strategy and concept would not only increase the bioavailability of SC for triggering the energy expenditure of BAT but also avoid possible systemic inflammation. Thus, a simple idea based on accelerating the energy dissipation of BAT for thermogenesis by locally and temporarily transdermal SC delivery may become a potential and effective strategy that can avoid obesity development.

## Supporting Information Available

Pit formation was found on the porcine skin after microneedle treatment; food intake and water consumption of HFD-fed mice treated with SCW or SCMN, local SCMN treatment, which reduced fat mass of HFD-fed mice; microneedle treatments, which did not change physiological demand of HFD-fed mice, model of superoxide formation by the SC in WT-1 cells, permeabilizer, which was necessary to quickly archive SC permeabilization in mature brown adipocytes WT-1 cells; relative mRNA expression of browning markers, lipid metabolism markers, and macrophage polarization gene markers in WAT of HFD-fed mice with different delivery routes of SC; serum lipid profiles of HFD-fed mice treated with SCW or SCMN; and primer sequences for qPCR (PDF).

## Abbreviations

CIDEA: cell death-inducing DFFA (DNA fragmentation factor-α)-Like Effector A
ETC: electron transport chain
PGC-1alpha: peroxisome proliferator-activated receptor-gamma coactivator (PGC)-1alpha
PRDM16: PR (PRD1-BF1-RIZ1 homologous)-domain-containing protein
TCA: tricarboxylic acid
