# Supplementary material for: Transdermal Delivery of Succinate Accelerates Energy Dissipation of Brown Adipocytes to Reduce Remote Fat Accumulation
Source: Mol Pharm. 2022 Oct 25;19(11):4299–310. doi: 10.1021/acs.molpharmaceut.2c00628 (PMC9644396; doi:10.1021/acs.molpharmaceut.2c00628)
Supplement: Supplementary file 1 — mp2c00628_si_001.pdf [file mp2c00628_si_001.pdf]

Transdermal Delivery of Succinate Accelerates Energy Dissipation of Brown Adipocytes to Reduce Remote Fat Accumulation

*Fang-Hsuean Liao, Chun-Nien Yao, Shu-Ping Chen, Te-Haw Wu, Shu-Yi Lin**

Institute of Biomedical Engineering and Nanomedicine, National Health Research Institutes. 35 Keyan Road, Zhunan Town, Miaoli County 35053, Taiwan

*Corresponding author. Institute of Biomedical Engineering and Nanomedicine, National Health Research Institutes. 35 Keyan Road, Zhunan Town, Miaoli County 35053, Taiwan. E-mail: shuyi@nhri.edu.tw

Supplementary Information

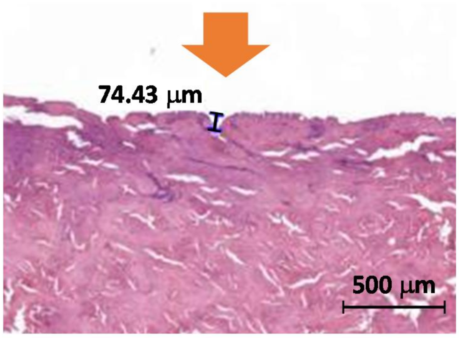

Figure S1. A pit formation was found on the porcine skin after microneedle treatment. The representative images of H&E stained cross-section of porcine skin after HA microneedle application. Scale bars, 500 μm.

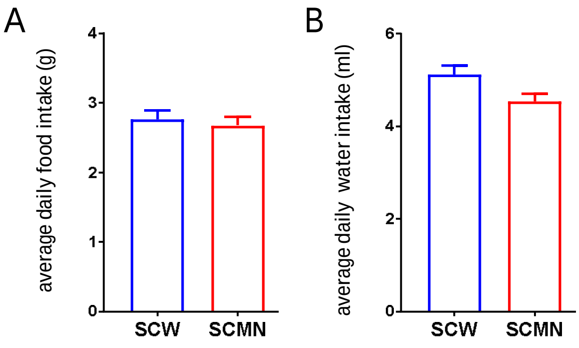

Figure S2. (A) Food intake and (B) water consumption of HFD-fed mice treated with succinate water (SCW) or succinate microneedle (SCMN) were recorded during the experiment. Data were expressed as mean ± SEM (n = 3–4).

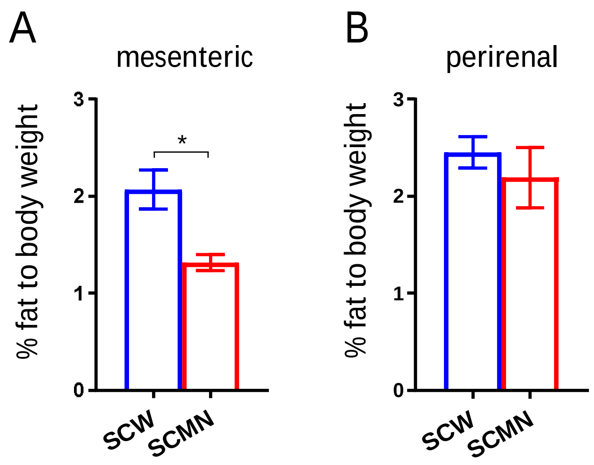

Figure S3. The local succinate microneedle (SCMN) treatment reduced fat mass of HFD-fed mice. The percentage of mesenteric (A) and perirenal (B) white adipose tissues against body weight of HFD-fed mice treated with succinate water (SCW) or SCMN. Data were expressed as mean ± SEM (n = 3–4). *$p < 0.05$ by unpaired, two-tailed Student's t-tests.

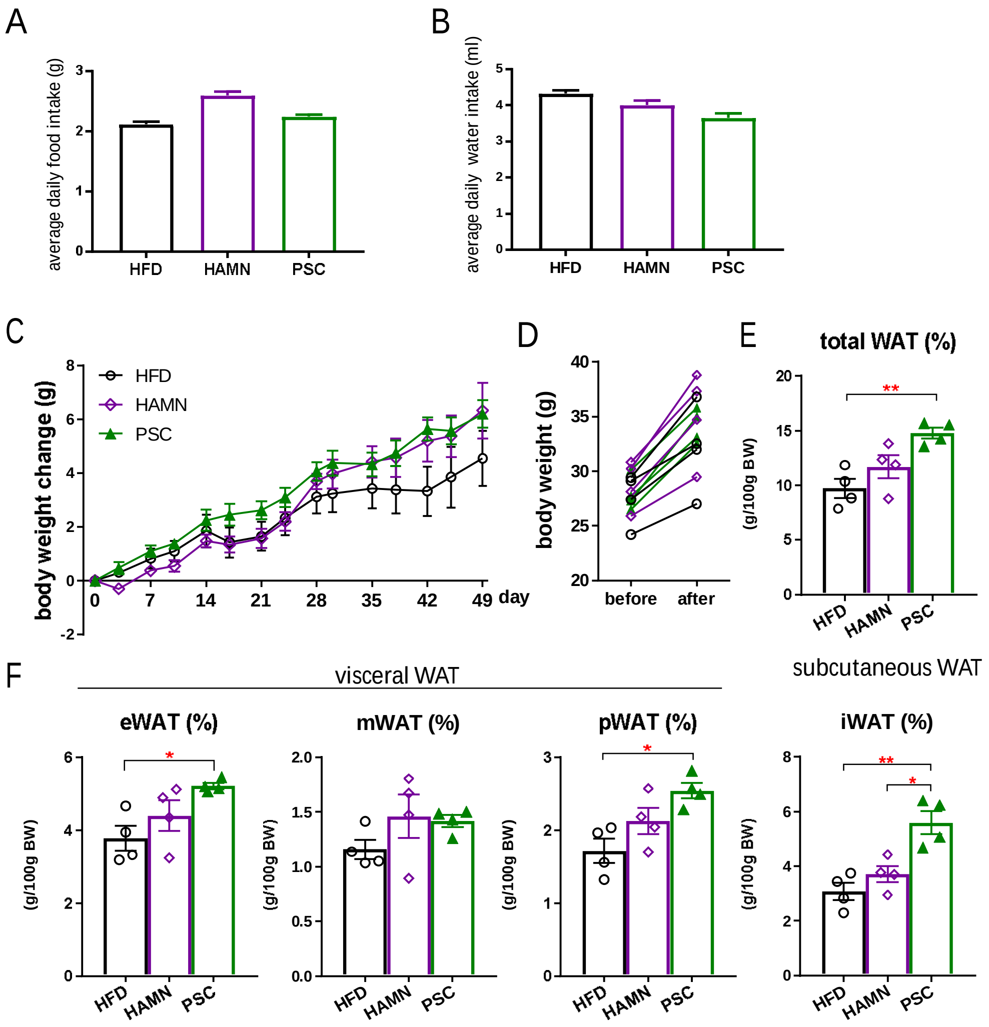

Figure S4. Microneedle treatments did not change physiological demand of HFD-fed mice. (A) Food intake, (B) water consumption, (C) body weight change, (D) body weight, and body fat mass of hyaluronic acid microneedle (HAMN) and plastic silicon microneedle (PSC) treatments of mice eating an HFD. Data were expressed as mean ± SEM ($n = 4$). *$p < 0.05$; **$p < 0.01$ by one-way ANOVA with Tukey's multiple comparisons.

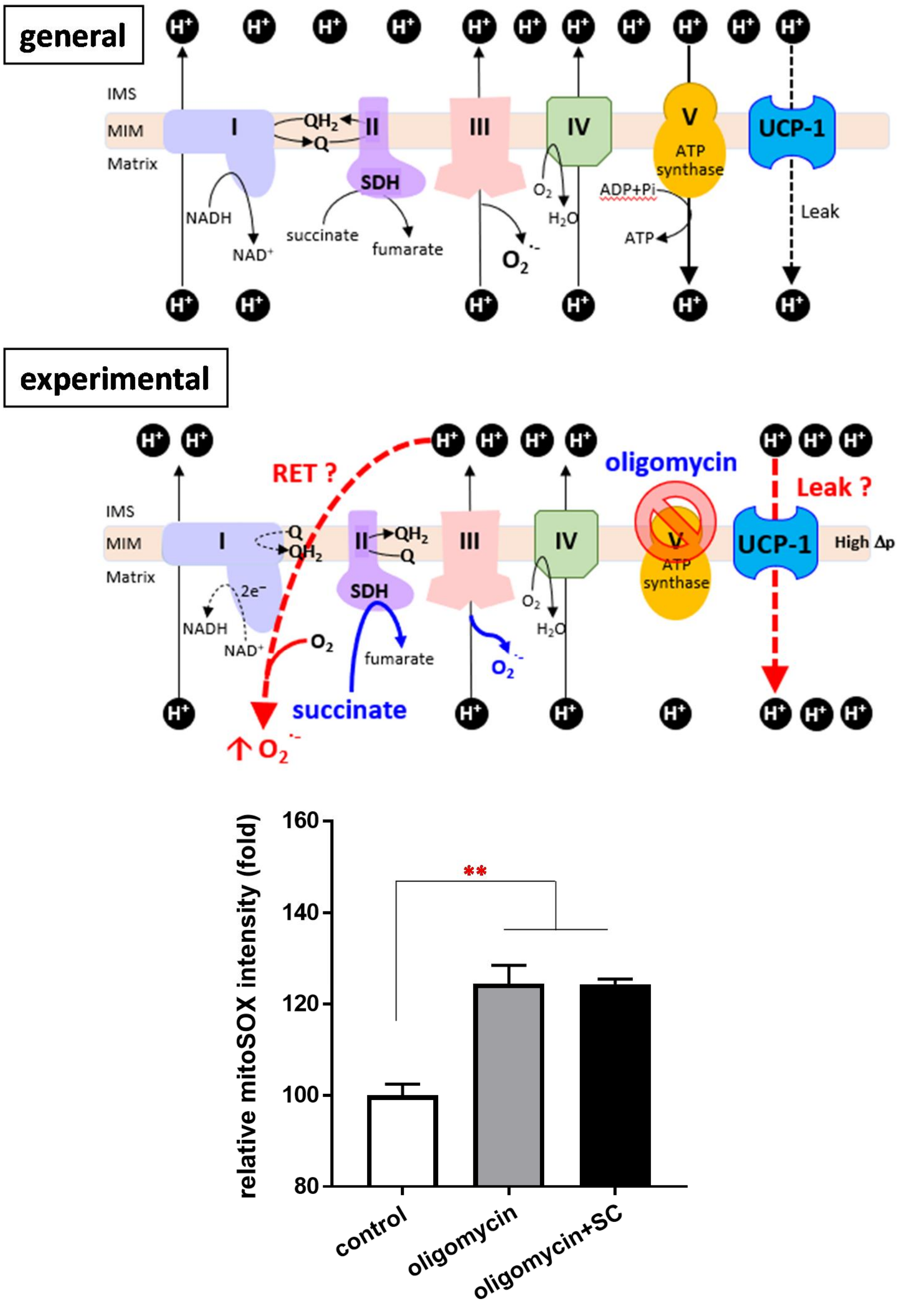

Figure S5. The model of superoxide formation by the succinate treatment in the presence of oligomycin excluded the reverse electron transfer contribution. Mitochondrial ROS levels of permeabilized mature brown adipocytes WT-1 cells treated with 1 μM oligomycin or additional 5 mM succinate (SC) for 30 min by mitoSOX staining. The upper panel represented the production

of mitochondrial superoxide anions by the electron transport chain of general condition. Oligomycin blocked ATP synthase leading to the increasing of proton gradient (high Dp) and ROS production. The middle panel illustrates the possible superoxide production by reverse electron transfer (RET) during the succinate addition in the presence of oligomycin (the left thick red dashed line) or the existence of an alternative pathway for proton leaks (the right thick red dashed line). WT-1 cells treated a combination with succinate and oligomycin did not boost mitochondrial ROS production. Abbreviations: IMS, intermembrane space; MIM, mitochondrial inner membrane; Q, ubiquinone; QH2, ubiquinol; SDH, succinate dehydrogenase; Dp, proton motive force. Data were expressed as mean ± SEM (n = 4–5). **p < 0.01 by one-way ANOVA with Tukey's multiple comparisons.

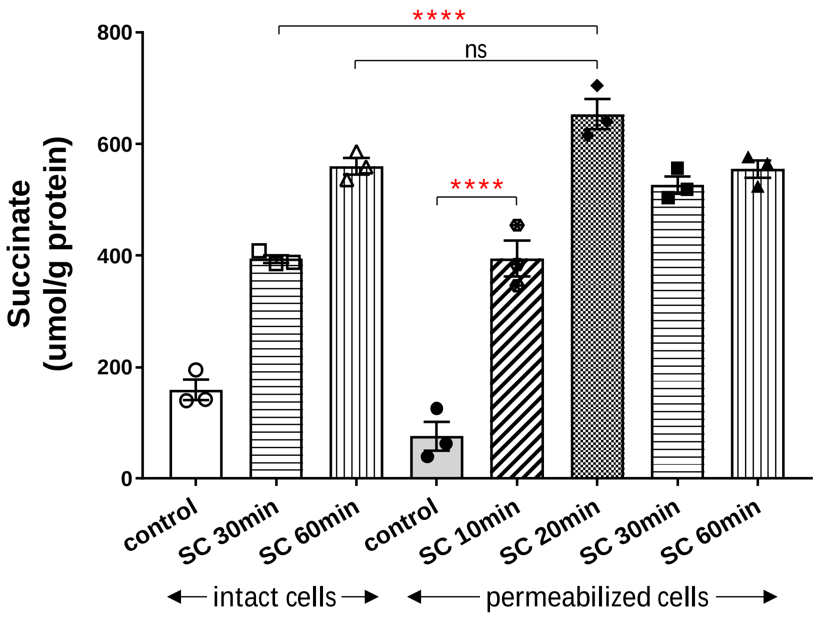

Figure S6. A permeabilizer was necessary to quickly archive succinate permearbilization in mature brown adipocytes WT-1 cells. Intracellular succinate levels of intact (non-permeabilized)

and permeabilized WT-1 cells treated 5 mM succinic-Na for the indicated time points. These results indicated that intact WT-1 cells spent at lesast 1 hr to reach the highest intracellular succinate concentrations due to a diffusion-controlled uptake. The permeabilized cells treated succinate for 10 mim reached the highest intracellular succinate concentrations comparable to the non-permeabilized cells for 30min. SC, succinate. Data were expressed as mean ± SEM (*n* = 3). ****$p < 0.0001$ by one-way ANOVA with Tukey's multiple comparisons and ns means no significance.

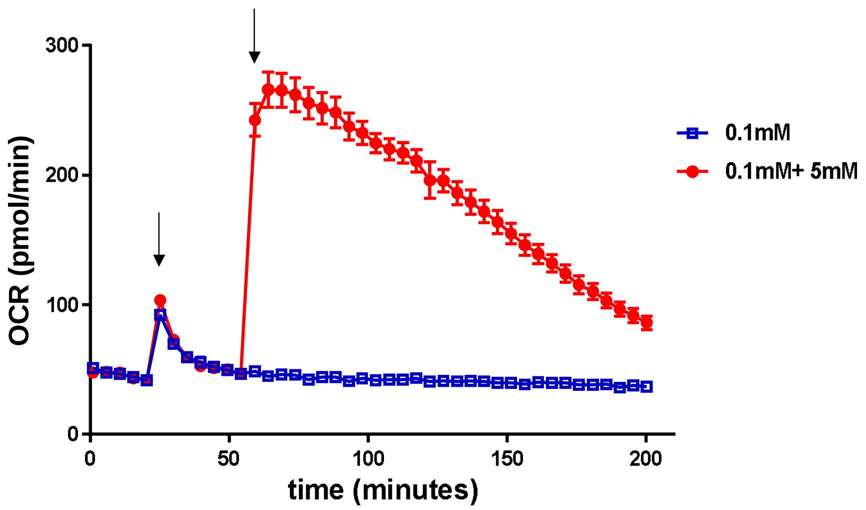

Figure S7. Succinate caused a concentration-dependent increase of mitochondrial respiration in WT-1 cells. Oxygen consumption rate (OCR) was measured on a Seahorse XF24 Analyzer under basal conditions and during serial addition of 0.1 mM and/or 5 mM succinate in permeabilized mature WT-1 cells. Arrows mean the addition of succinate. Data were expressed as mean ± SEM (*n* = 4–5).

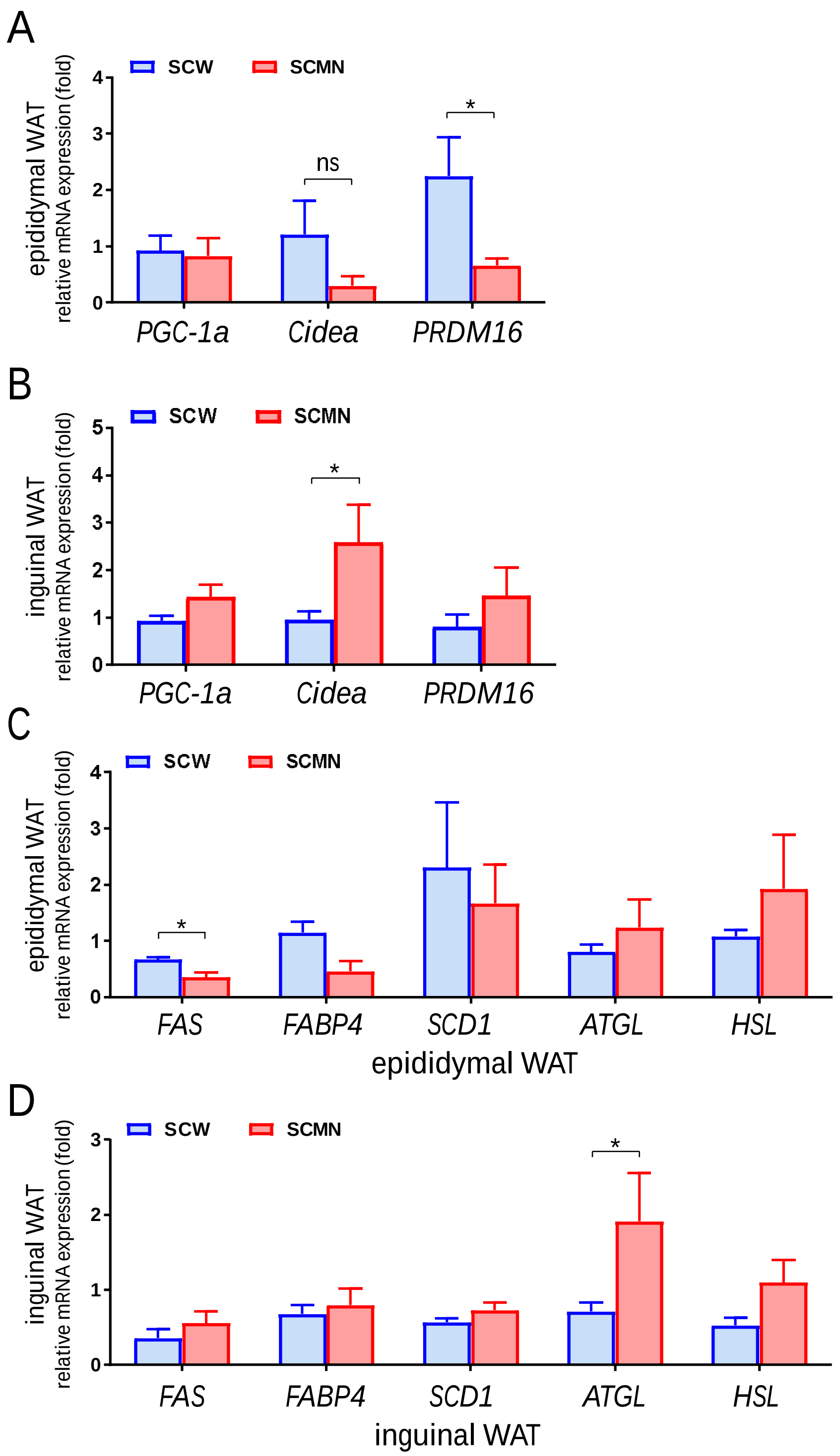

Figure S8. The relative mRNA expression of browning markers (A and B) and lipid metabolism markers (C and D) in WAT of HFD-fed mice with different delivery routes of succinate was

validated. The relative mRNA expression of in epididymal (A and C) and inguinal (B and D) white adipose tissue of HFD-fed mice treated with succinate water (SCW) or succinate microneedle (SCMN) is presented relative to those of untreated HFD-fed mice. Data were expressed as mean ± SEM (n = 3–4). *$p < 0.05$ by unpaired, two-tailed Student's t-tests.

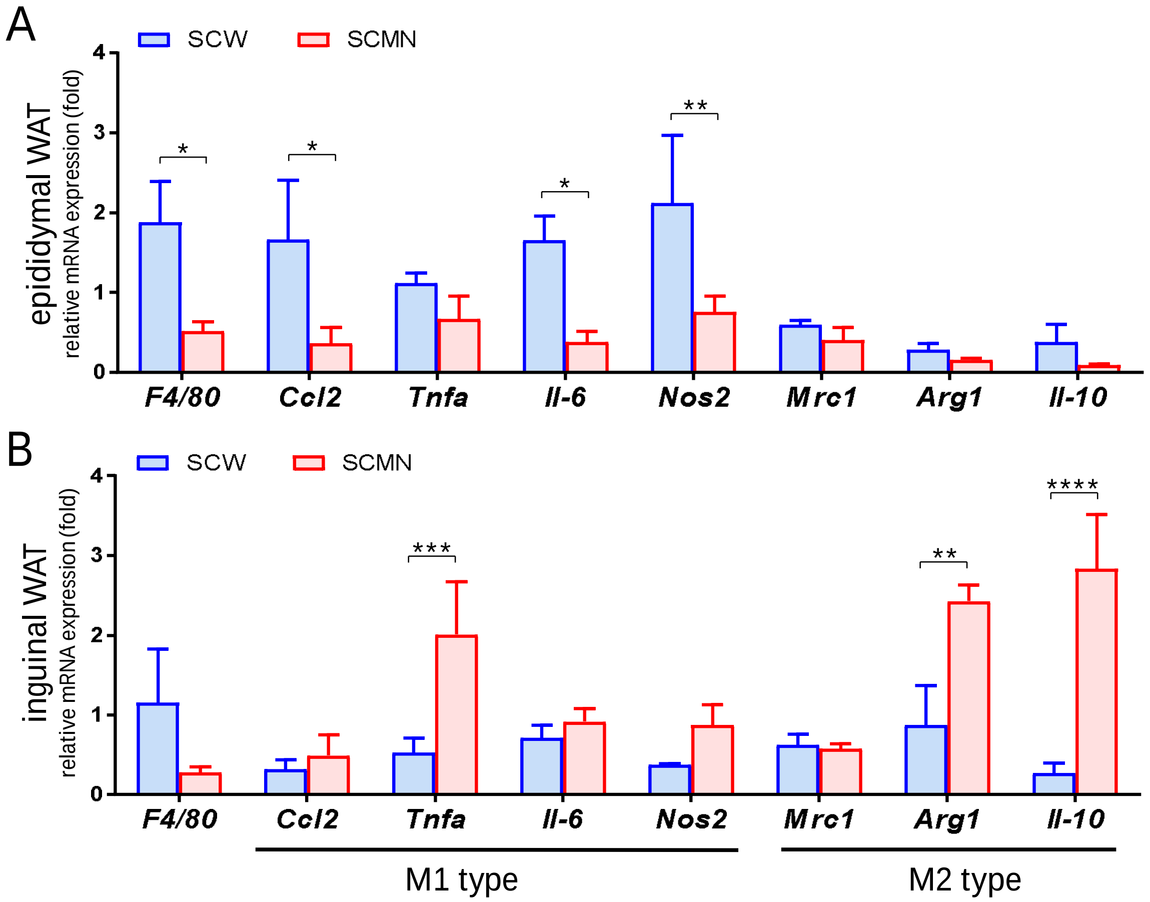

Figure S9. The relative mRNA expression of macrophage polarization gene markers in WAT of HFD-fed mice with different delivery routes of succinate. The relative mRNA expression of respective macrophage polarization markers in (A) epididymal and (B) inguinal white adipose tissue of HFD-fed mice treated with succinate water (SCW) or succinate microneedle (SCMN) is

presented relative to those of untreated HFD-fed mice. *Ccl2* is abbreviated from C-C motif chemokine ligand 2, also known as monocyte chemoattractant protein-1 (MCP-1). *Nos2* encodes nitric oxide synthase 2, *Mrc1* encodes the mannose receptor CD206, *Arg1* encodes the ureohydrolase arginase. Data were expressed as mean ± SEM (n = 3–4). *$p < 0.05$; **$p < 0.01$; ***$p < 0.001$; ****$p < 0.0001$ by unpaired, two-tailed Student's t-tests.

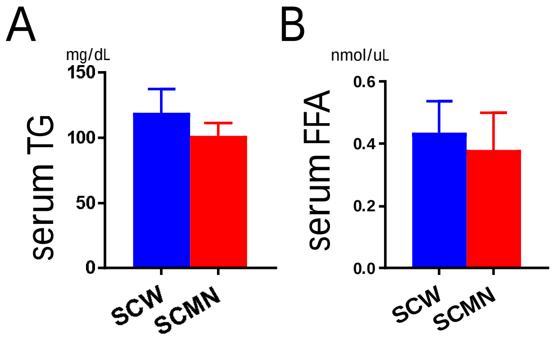

Figure S10. The serum lipid profiles of HFD-fed mice treated with succinate water (SCW) or succinate microneedle (SCMN). (A) Serum triglyceride (TG) and (B) serum free fatty acid (FFA) concentrations of mice with different treatments were measured. Data were expressed as mean ± SEM (n = 3–4).

Supplementary Table 1. Primer sequences for qPCR.

name	primer	Oligonucleotides 5‘-3‘ sequences
UCP1	Forward	aagggtttgtggcttcttttct
	Reverse	tgtctgtctggacttcatcagc
PGC-1a	Forward	gaaaaagaagtcccatacacaacc
	Reverse	cagttccagagagttccacactt
Cidea	Forward	ttaagggacaacacgcatttca
	Reverse	ttcccgatttctttggttgctt
PRDM16	Forward	caggaaggggaaggagagatac
	Reverse	gaggttggaggagatgctga
FAS	Forward	cagatgatgacaggagatggaa
	Reverse	attgtgtgtgcctgcttgg
FABP4	Forward	cagacgacaggaaggtgaaga
	Reverse	gctcatgccctttcataaactc
SCD1	Forward	gacctgaaagccgagaagc
	Reverse	ccagagtgtatcgcaagaagg
ATGL	Forward	ccactttagctccaaggatgag
	Reverse	cctgagaatggggacactg
HSL	Forward	ctcatggaccctcttctaccac
	Reverse	catcacgcctagtgccttc
F4/80	Forward	tgcagtgtcagctcagaagt
	Reverse	gtatgccatgatgcttgcca
Ccl2	Forward	ccactcacctgctgctact
	Reverse	ctcacactacagcttctttggg
Tnfa	Forward	ccaccacgctcttctgtcta
	Reverse	agctgctcctccacttggt

Il-6	Forward	tgccttcttgggactgatg
	Reverse	gccattgcacaactcttttct
Nos2	Forward	gagtcttggtgaaagtggtgttc
	Reverse	ttggtgttgaaggcgtagc
Mrc1	Forward	ggatggatgggagcaaagt
	Reverse	gtcgtgggtgtggtaggc
Arg1	Forward	gtggggaaagccaatgaa
	Reverse	ttgtcaggggagtgttgatg
Il-10	Forward	tcggaaatgatccagttttacc
	Reverse	ctccactgccttgctcttattt
CPT1a	Forward	tctggatgcggtagaaaagg
	Reverse	ttgaagacaacaaaggtgatgg
Beta-actin	Forward	aagtgtgacgttgacatccgtaa
	Reverse	tgcctgggtacatggtggta
36B4	Forward	gtcctggcattgtctgtgg
	Reverse	tccgactcttcctttgcttc
